# Giant Arachnoid Cyst Presenting With Transient Facial Paresthesia: A Case Report

**DOI:** 10.7759/cureus.103713

**Published:** 2026-02-16

**Authors:** Ana Maria Carvalho, Pedro Sá Almeida, Rita G Magalhães, Marta Madarnas, Beatriz Exposito

**Affiliations:** 1 Internal Medicine, Unidade Local de Saúde de Trás-os-Montes e Alto Douro, Chaves, PRT

**Keywords:** arachnoid cyst, brain imaging, case report, computed tomography, conservative management, intracranial cyst

## Abstract

Arachnoid cysts are benign, cerebrospinal fluid-filled lesions that are frequently discovered incidentally on neuroimaging. Although most remain asymptomatic, large cysts may occasionally be associated with neurological symptoms due to mass effect or compression of adjacent structures.

We report the case of a 61-year-old man who presented with transient numbness of the left side of the face. Brain computed tomography (CT) revealed a large right parieto-occipital arachnoid cyst with cerebrospinal fluid-like density, smooth margins, and thinning of the overlying calvaria, consistent with a long-standing lesion. The patient’s symptoms resolved spontaneously, and no additional neurological deficits were observed during follow-up.

This case illustrates the frequent dissociation between the striking radiologic appearance of giant arachnoid cysts and their limited clinical expression. Despite its size, the lesion was associated only with mild and self-limited symptoms, supporting a conservative management approach.

Giant arachnoid cysts may remain clinically silent or present with transient symptoms. Clinical management should be guided by symptom severity and evolution rather than imaging findings alone in order to avoid unnecessary interventions.

## Introduction

Arachnoid cysts are cerebrospinal fluid-filled cavities that develop between the arachnoid membranes covering the brain or spinal cord. They are benign lesions, most commonly of congenital origin, although acquired forms have also been described [1,2]. The estimated prevalence in the general population ranges from 1% to 2%, with a marked male predominance, reported to be approximately two to four times higher than in females [1,3].

In most cases, arachnoid cysts are identified incidentally on neuroimaging studies performed for unrelated reasons and remain asymptomatic throughout life [1,4]. However, cyst enlargement or mass effect on adjacent neural structures may lead to neurological manifestations, including hydrocephalus, seizures, or focal neurological deficits, potentially prompting medical or surgical intervention [5,6]. In the absence of significant or progressive symptoms, management is typically conservative, with clinical and imaging surveillance being the preferred approach [3,4]. Although there is no universally accepted definition, arachnoid cysts are often described as “giant” when they occupy a substantial portion of a cerebral hemisphere or produce a marked mass effect on adjacent structures. While most arachnoid cysts remain asymptomatic, focal sensory symptoms such as isolated facial paresthesia are uncommon presentations, making this case clinically noteworthy [1].

## Case presentation

We report the case of a 61-year-old man, autonomous and cognitively intact, with a history of well-controlled type 2 diabetes mellitus, who presented to the emergency department with left-sided facial paresthesia lasting three days. The symptoms had a gradual onset, were not associated with headache, weakness, speech disturbance, or visual changes, and had occurred in similar self-limited episodes in the past without identifiable triggers.

**Figure 1:**
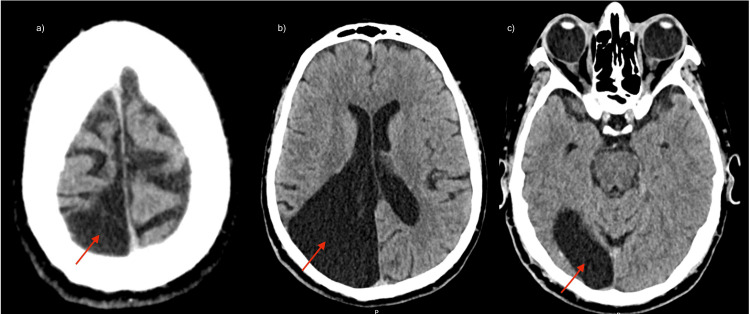
Axial brain computed tomography images showing a large right parieto-occipital arachnoid cyst. a–c: large cystic lesion (arrows) with attenuation similar to cerebrospinal fluid, showing smooth and well-defined margins, located in the right parieto-occipital region.

**Figure 2:**
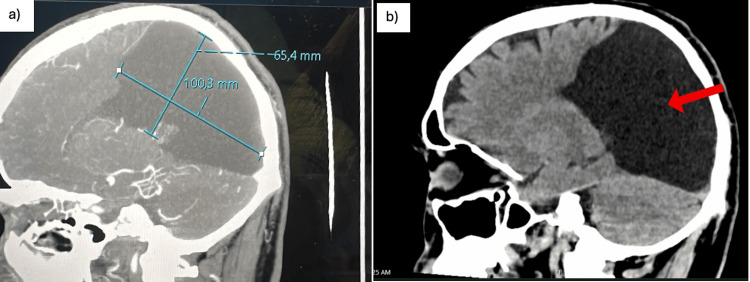
Sagittal brain computed tomography images illustrating the craniocaudal extent of the right parieto-occipital arachnoid cyst. a: axial brain CT image showing measurement of the right parieto-occipital arachnoid cyst in maximal dimensions (100.3 x 65.4 mm); b: sagittal view demonstrating the craniocaudal extent of the cyst (arrow) and chronic remodeling of the adjacent calvaria.

**Figure 3:**
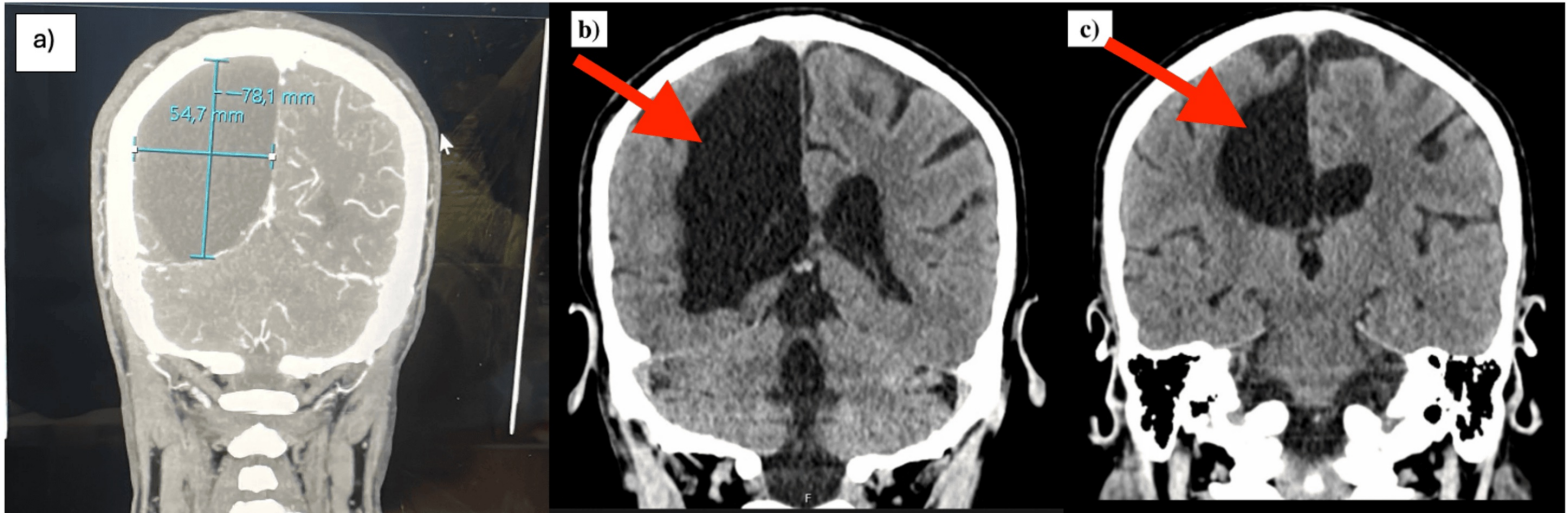
Coronal brain computed tomography images demonstrating the size and location of the right parieto-occipital arachnoid cyst. a: coronal brain CT image showing measurement of the right parieto-occipital arachnoid cyst in maximal dimensions (54.7 x 78.1 mm); b,c: coronal views showing the right parieto-occipital cyst (arrows) with preservation of midline structures and absence of surrounding edema.

Neurological examination revealed left facial hypoesthesia, with no other focal neurological deficits. Brain computed tomography (CT) showed a large arachnoid cyst involving the right cerebral hemisphere, measuring approximately 10.04 x 7.81 x 5.47 cm in maximal dimensions, with imaging features suggestive of a chronic lesion and no evidence of acute complications (Figures 1-3).

The case was discussed with the neurosurgery team, who considered outpatient follow-up appropriate given the absence of signs of intracranial hypertension and the mild, self-limited nature of the symptoms. The neurology team recommended clinical observation to document any potential recurrence or associated involuntary movements. Magnetic resonance imaging was considered; however, given the typical CT appearance and the patient’s stable clinical condition, conservative management was prioritized.

During hospitalization, the patient experienced complete resolution of the facial hypoesthesia, with no recurrence or new neurological findings. At one-year follow-up, the patient remained asymptomatic, and repeated CT imaging demonstrated a stable appearance of the arachnoid cyst without interval growth or new findings.

## Discussion

Arachnoid cysts are cerebrospinal fluid collections located between the arachnoid membranes and may be congenital or acquired [1,4]. Most are discovered incidentally during neuroimaging, although large cysts may become symptomatic due to compression of adjacent structures [1,2]. Clinical manifestations depend on cyst size and location and can include headache, focal neurological deficits, seizures, or signs of intracranial hypertension [4,6].

CT is often sufficient for diagnosis, typically demonstrating a well-defined hypodense lesion with cerebrospinal fluid attenuation and no contrast enhancement. Magnetic resonance imaging may be useful to confirm the diagnosis and to exclude alternative entities such as epidermoid cysts or cystic neoplasms [6]. In the present case, CT findings were consistent with a long-standing lesion, without features suggestive of an acute complication.

Management is generally conservative in asymptomatic patients or in those with mild and self-limited symptoms. Surgical intervention is reserved for cases with significant mass effect, persistent symptoms, or progressive neurological deterioration [3,4]. Available surgical options include endoscopic fenestration, microsurgical fenestration, and cystoperitoneal shunting. Although endoscopic approaches are less invasive, they may be associated with a higher risk of recurrence [3,5].

Several conditions may account for isolated facial hypoesthesia, many of which are self-limited and without a clearly identifiable etiology. In the present case, a definitive causal relationship between the arachnoid cyst and the facial sensory symptoms cannot be established, particularly given the transient nature of the presentation and the absence of recurrence during the one-year follow-up. The cyst was therefore considered an incidental imaging finding, most likely congenital in origin. Nevertheless, the considerable size of the lesion without associated severe neurological deficits remains noteworthy.

## Conclusions

Arachnoid cysts are frequently incidental findings but may occasionally be associated with transient neurological manifestations. In this case, despite the large dimensions of the lesion, the clinical presentation was mild and self-limited. The absence of a consistent correlation between cyst size and symptom severity underscores the importance of an individualized and cautious approach to management and follow-up.
